# Tixagevimab and Cilgavimab (Evusheld) Boosts Antibody Levels to SARS-CoV-2 in End-Stage Renal Disease Patients on Chronic Hemodialysis: A Single-Center Study

**DOI:** 10.3390/medicina59122109

**Published:** 2023-12-01

**Authors:** Mohammed Kamal Nassar, Alaa Sabry, Mohamed Elgamal, Zeinab Zeid, Dalia Abdellateif Abdelghany, Samar Tharwat

**Affiliations:** 1 Mansoura Nephrology & Dialysis Unit (MNDU), Department of Internal Medicine, Faculty of Medicine, Mansoura University, Mansoura 35516, Egypt; m_kamal@mans.edu.eg (M.K.N.); asabry2040@mans.edu.eg (A.S.); 2 Department of Internal Medicine, Faculty of Medicine, Horus University, New Damietta 34517, Egypt; 3 Chest Department, Faculty of Medicine, Mansoura University, Mansoura 35516, Egypt; melgamal6@gmail.com (M.E.); daliaabdellateif@mans.edu.eg (D.A.A.); 4 Al-Khezam Dialysis Center, Al-Adan Hospital, Hadiya 47000, Kuwait; zainabzaid78@gmail.com; 5 Rheumatology & Immunology Unit, Department of Internal Medicine, Faculty of Medicine, Mansoura University, Mansoura 35516, Egypt

**Keywords:** tixagevimab, cilgavimab, Evusheld, hemodialysis

## Abstract

*Background and Objectives*: In addition to a suboptimal and rapidly diminishing response to the coronavirus disease 2019 (COVID-19) vaccine, hemodialysis (HD) patients are at risk for developing a severe COVID-19 infection. In 2022, the combination of cilgavimab and tixagevimab (Evusheld, AstraZeneca) was approved for COVID-19 preexposure prophylaxis in high-risk groups. The purpose of this study was to evaluate the humoral response and short-term safety of this antibody combination in a group of HD patients. *Materials and Methods*: Seventy-three adult maintenance hemodialysis patients were recruited from a tertiary-care hospital for this double-blinded, non-randomized, placebo-controlled study. Patients were placed into two groups: the intervention group (n = 43) received a single 300 mg dosage of cilgavimab and tixagevimab, while the control group (n = 30) received a saline placebo. The titer of COVID-19-neutralizing antibodies was measured at baseline and after 1 and 6 months. The patients were evaluated for any drug-related adverse effects and monitored for six months for the emergence of any COVID-19-related events. *Results*: Patients in the intervention group were substantially older and had been on HD for longer (*p* = 0.002 and 0.006, respectively). The baseline antibody levels were higher in the Evusheld group. The antibody level in the intervention group increased significantly after 1 month and remained consistent for 6 months, whereas the antibody level in the control group fell significantly after 6 months during the study period (Wald χ^2^ = 30.620, *p* < 0.001). The drug-related adverse effects were modest and well-tolerated, and only seven patients experienced them. Six months after study enrollment, 10 patients in the intervention group and 6 patients in the control group had been infected with COVID-19, respectively. In the control group, ICU admission and mortality were observed, but in the intervention group, the infection was milder with no aggressive consequences. *Conclusions*: This study demonstrated the short-term safety and efficacy of tixagevimab–cilgavimab for COVID-19 preexposure prophylaxis in HD patients. These findings require more studies with more HD patients and longer follow-up periods.

## 1. Introduction

Coronavirus disease 2019 (COVID-19) is caused by the single-stranded RNA virus that causes severe acute respiratory syndrome coronavirus 2 (SARS-CoV-2). It was first discovered in December 2019 in Wuhan, China, and has since spread fast around the globe. On 11 March 2020, a global pandemic was declared [1,2]. By 27 October 2023, the World Health Organization (WHO) estimated that there were more than 771 million confirmed COVID-19 cases and 6,974,460 deaths globally [3]. 

There was a significant association observed between underlying chronic conditions and both heightened COVID-19 disease severity and increased admission to the intensive care unit (ICU) [4]. Chronic kidney disease (CKD) patients on maintenance hemodialysis (HD) are a high-risk category for the contraction of COVID-19, with reported significant fatality rates [5]. This is due to comorbidities such as diabetes mellitus, hypertension, cardiovascular disease, advanced age, and the existence of uremia-induced immune suppression and proinflammatory conditions [6]. 

Throughout the successive pandemic waves, infection control methods were gradually applied in dialysis centers to reduce the transmission rate [7,8]. In addition, COVID-19 vaccinations appeared effective in lowering hospitalizations and severe illness [9]. Therefore, the COVID-19 vaccination is recommended for HD patients. Even though the COVID-19 vaccines have a high acceptance rate [10] and a good short-term safety profile among HD patients [11,12,13], these patients are more likely to have a suboptimal vaccine response, with weaker and waning humoral responses compared to healthy controls [14,15].

There is a positive correlation between the serum’s potential to neutralize SARS-CoV-2 and protection against COVID-19 [16]. In the United Kingdom, the combination of cilgavimab and tixagevimab (Evusheld, AstraZeneca, Cambridge, UK) was approved in March 2022 for the protection of transplant recipients who had an inadequate response to vaccination against the COVID-19 infection [17]. The phase-III double-blind, placebo-controlled study of AZD7442 for Preexposure Prophylaxis of COVID-19 in Adults (PROVENT) demonstrated the efficacy of a single dose of 300 mg of cilgavimab–tixagevimab with no obvious safety issues [18]. Nevertheless, there is currently a lack of research papers that have provided evidence regarding its effectiveness and safety for individuals undergoing HD. 

In this study, the neutralizing capacity and short-term safety of cilgavimab and tixagevimab against COVID-19 infection were examined in a cohort of Egyptian chronic HD patients who received the medication for preexposure prophylaxis.

## 2. Materials and Methods

### 2.1. Patients

Seventy-three adult HD patients (age > 18) were recruited for this double-blind, non-randomized, placebo-controlled interventional study at the Dialysis Unit of Mansoura University Hospitals, Egypt. All participants had been receiving HD treatment for more than six months. Exclusion criteria included patients with a recent COVID-19 infection within 1 month of trial initiation, a family or current history of immune deficiency problems, or those who refused to participate. At least three months before the study, a COVID-19 vaccination was administered to all the recruited patients. The enrolled patients were divided into two groups: the intervention group (group A) (*n* = 43) received a single 300 mg dose of the combination of cilgavimab and tixagevimab (Evusheld, AstraZeneca) (one 1.5 mL intramuscular injection of each antibody administered consecutively), and the control group (group B) (*n* = 30) received a saline placebo (two 1.5 mL intramuscular injections administered consecutively). The study was not randomized, as patients were assigned to the intervention group based on the priority indication due to the drug’s restricted availability. Those who were predicted to have a deficient humoral response to the vaccine and at high risk of severe COVID-19 infection, such as the elderly, diabetics, those using immunosuppressive drugs, and those with many comorbidities, were selected to receive the treatment. The Institutional Research Board of the Faculty of Medicine at Mansoura University authorized the study protocol (approval number: R.22.07.1769). Before beginning the trial, all patients were briefed on the purpose of the research, and they all provided signed consent.

Patients’ demographic information, including age and gender, was gathered. In addition, clinical variables such as duration since starting HD, related comorbidities, and treatment data were documented.

### 2.2. Blood Sampling and Laboratory Tests

Before the first HD session of the week, a total of 5 mL of whole blood was collected from the arteriovenous access of each participant on the same days of clinical assessment. The blood samples were collected in EDTA-free sterile tubes to obtain serum. Then, the serum was separated via centrifugation and stored at −20 °C.

### 2.3. COVID-19 Neutralization Antibody Titer Assessment

The titer of COVID-19-neutralizing antibodies was assessed at baseline and after 1 and 6 months using the iFlash-2019-nCoV neutralization antibody (Nab) assay (iFlash 1800 Chemiluminescence Immunoassay Analyzer Consolidation for Automation, Shenzhen Yhlo Biotech Co., Shenzhen, China). The iFlash-2019-nCoV Nab test is a competitive immunoassay utilizing CLIA as follows:

Briefly, the serum samples were placed on a sample rack in the sample loading area, and a reagent pack with 2019-nCoV Receptor Binding Domian (RBD) antigen (30KD)-coated paramagnetic microparticles and acridinium ester-labeled Angiotensin Converting Enzyme 2 (ACE2) conjugate was placed in the reagent loading area. Under a magnetic field, magnetic particles were adsorbed to the wall of the reaction tube, and unbound materials were washed away using the wash buffer. The pre-trigger and trigger solutions were added to the reaction mixture. The resulting chemiluminescent reaction was measured in relative light units (RLUs). Using the ROC curve approach, the cut-off value of 10.00 arbitrary units (AU)/mL was determined for the iFlash-2019-nCoV Nab reagent set. The results were obtained using a calibration curve that was constructed using a four-point calibration and a master calibration curve provided via the QR code on the reagent. The range of measurement was 4–800 AU/mL. At 4 AU/mL, values below the lower limit of the measurement range were recorded. More than 800 AU/mL was used to report values above the measurement range.

### 2.4. End Points

#### 2.4.1. Safety End Point

The primary safety goal was to determine the incidence of adverse events following the intramuscular administration of a single dosage of cilgavimab–tixagevimab as compared to the placebo.

#### 2.4.2. Efficacy End Point

The primary efficacy end point was the occurrence of any of the following COVID-19-related outcomes:

- Asymptomatic SARS-CoV-2 infection: SARS-CoV-2 infection verified by either a molecular or antigen test.
- Symptomatic COVID-19 disease: SARS-CoV-2 infection confirmed by a molecular or antigen test and at least one of the following symptoms at the time of testing: fever, shortness of breath, difficulty breathing, new-onset confusion (only for participants ≥60 years), loss of appetite or decrease in food intake (only for participants ≥60 years on baseline supplemental oxygen), cough, fatigue, headache, body aches, runny nose, nausea, vomiting, and diarrhea.
- COVID-19-related hospitalization: Patients with a recorded COVID-19 infection who required hospitalization due to the COVID-19 infection.
- ICU admission for COVID-19: An ICU admission or discharge note indicating that COVID-19 was the patient’s primary diagnosis.
- COVID-19-related death: Death certificates indicating that COVID-19 was a contributing factor in the decedent’s passing; hospitalization and subsequent death with COVID-19 indicated as the primary diagnosis; or death within 28 days of a COVID-19 diagnosis and hospitalization.

### 2.5. Statistical Analysis

SPSS (Statistical Package of Social Sciences) version 21 for Windows (SPSS, Inc., Chicago, IL, USA) was used to conduct statistical analysis. Numbers and percents were used to describe quantitative data (n, %). When applicable, the data were first evaluated for normality using the Shapiro–Wilk test or the Kolmogorov–Smirnov test. For normally distributed data, the mean ± standard deviation (SD) was used, and for non-normally distributed data, the median (interquartile range) was used. When comparing two groups with quantitative normally distributed data, the Independent Samples *t*-test was used, whereas when comparing two groups with quantitative non-normally distributed data, the Mann–Whitney test was employed. When comparing qualitative data with a 2 × 2 table, the chi-square or Fisher’s exact test was applied. Univariate correlation analysis was carried out with the Pearson test for normally distributed data and the Spearman test for non-normally distributed variables. A statistically significant *p* value was less than 0.05.

## 3. Results

The current study included 73 HD patients with a median age of 48 years and a majority (63%) of males. More than half of the patients had hypertension (60.3%), and 16.4% had diabetes. The study groups’ sociodemographic, clinical, and therapeutic characteristics are displayed in Table 1. Group A patients were significantly older and had been receiving HD for a longer duration (*p* = 0.002 and 0.006, respectively) (Table 1).

In terms of previous COVID-19 infections, 26% of patients had a history of infection. All the patients received the vaccine, and more than three-quarters received the SINOVAC vaccine. In terms of past COVID-19 infection and vaccination, there was no significant difference between the two trial groups, with a median interval of 145 days between the administration of the vaccine and cilgavimab–tixagevimab (Table 2).

Based on the non-Gaussian distribution of the NAB titer values, the values were categorized into three categories according to the first and third quartiles (200 and 800 AU/mL, respectively). The baseline NAB scores of individuals who did not receive Evusheld were significantly higher than those who did receive Evusheld (median score 3 vs. 2). Those who received Evusheld had higher NAB scores at one and six months compared to those who did not (median scores of 3 versus 2, and 3 versus 1.5, respectively) (Table 3). A factorial ordinal regression model using Generalized Estimating Equations (GEE) with Evusheld use and time as predictors and NAB score as a dependent variable revealed that NAB score differs significantly between those with and without Evusheld use (Wald χ^2^ [1] = 22.538, *p* < 0.001). Also, time (Wald χ^2^ [2] = 8.982, *p* = 0.011) and group–time interaction (Wald χ^2^ [2] = 30.620, *p* < 0.001) affected the NAB score significantly.

Approximately one-fifth of both groups were diagnosed with COVID-19 throughout the six-month trial period, but the severity was greater in the control groups. Three of the six patients in the control group with a confirmed COVID-19 infection required ICU care, while the other three patients died from COVID-19 infection. Comparatively, of the 10 patients in the intervention group who contracted an infection, 4 had asymptomatic infections and 6 required hospitalizations, but neither ICU admission nor mortality occurred (Table 4). 

**Figure 1:**
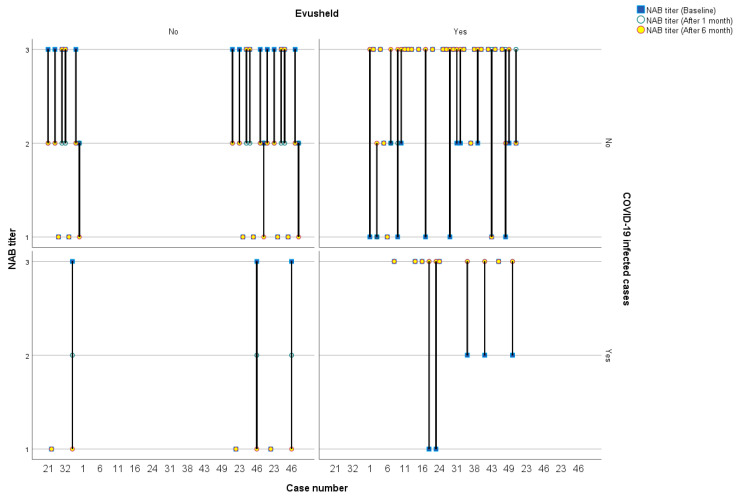
COVID-19-neutralizing antibody titer scores for individual patients at various time periods categorized by COVID-19 infection status and intervention group.

The NAB scores for individual patients at various time periods are depicted in Figure 1, with the lines representing these scores categorized by COVID-19 infection status and intervention group.

Regarding the short-term safety endpoint, only seven patients reported mild, well-tolerated side effects, none of which needed hospitalization (Table 5). 

## 4. Discussion

In this double-blind, non-randomized, placebo-controlled interventional trial, the short-term safety and immunogenicity of tixagevimab–cilgavimab against COVID-19 infection were evaluated in end-stage renal disease (ESRD) patients on chronic HD. The titer of COVID-19-neutralizing antibodies was assessed at baseline and after 1 and 6 months. Antibody levels significantly increased one month after tixagevimab–cilgavimab injection and remained steady for six months. The adverse effects of tixagevimab–cilgavimab were mild, well-tolerated, and did not require hospitalization. Within six months of taking tixagevimab–cilgavimab, approximately one-fifth of patients developed a COVID-19 infection, which was less severe than in patients who did not receive tixagevimab–cilgavimab and was not associated with ICU admission or mortality.

Patients with HD are prone to COVID-19 due to their impaired immune systems and hospitalizations for life-sustaining treatment [19]. Twenty-six percent of participants in the current study had a confirmed COVID-19 infection. The number of patients with ESRD is increasing as the population ages, and the burden of HD patients in nephrology clinics has grown [20,21]. During the COVID-19 pandemic, the management and follow-up of HD patients posed a challenge for nephrologists [22]. 

Preexposure preventive treatment with monoclonal antibodies considerably reduced the occurrence of serious COVID-19 infection [3]. In our study, antibody levels significantly increased one month after tixagevimab–cilgavimab injection and remained steady for six months. The PROVENT (Prophylaxis Prevention) study [5] also concluded that the efficacy of tixagevimab–cilgavimab would last at least six months; however, the study was carried out before the Omicron era. The results of a recent randomized trial support the use of a single intramuscular dose of tixagevimab–cilgavimab for the prevention of symptomatic and severe COVID-19 [18]. Bertrand and colleagues showed the potential clinical value of tixagevimab–cilgavimab preexposure prophylaxis against the Omicron variant in kidney transplant recipients [23].

In the current study, the antibody level increased dramatically one month after tixagevimab–cilgavimab injection and remained stable for the following six months. In contrast, the titer of neutralizing antibodies in the non-tixagevimab–cilgavimab group decreased significantly during the same time period. The current findings seem to agree with the PROVENT (Prophylaxis Prevention) experiment, which estimated that tixagevimab–cilgavimab efficacy would remain for at least six months [18]. In a study with 98 kidney transplant recipients, however, the anti-receptor-binding domain of the SARS-CoV-2 spike protein IgG level dropped quickly after tixagevimab–cilgavimab was given [24]. This may be explained by the effect of the immunosuppressive medications on the body’s humoral response to vaccines.

In the current study, only seven individuals in the tixagevimab–cilgavimab group experienced mild, well-tolerated adverse effects, none of whom required hospitalization. Based on limited experience and the approvals in the United States and the United Kingdom, tixagevimab–cilgavimab may be a crucial product for delivering COVID-19 protection to patients who have previously experienced severe adverse reactions to the COVID-19 vaccine. Moreover, increased data collection and reporting are urgently required due to the scarcity of data [25]. During or after administration, tixagevimab–cilgavimab can cause allergic reactions, with symptoms including shortness of breath; chest pain; hives; wheezing; and swelling of the cheeks, lips, mouth, and tongue. Patients with underlying cardiac risk factors have been documented to experience infrequent major cardiac episodes [26]. Possible adverse effects of tixagevimab–cilgavimab therapy include injection-site pain, discomfort, swelling, bruising, and skin infection. Rarely, patients with cardiac risk factors who received tixagevimab–cilgavimab encountered serious cardiac side effects [26]. 

In the present study, approximately one-fifth of patients acquired COVID-19 infection within six months in both groups, which was less severe than in the non- tixagevimab–cilgavimab group, and it was not associated with ICU admission or mortality. It was hypothesized that monoclonal antibodies used as a preventative approach reduce the incidence of serious infections in immunocompromised people by a substantial amount [24]. According to Bertrand et al., tixagevimab–cilgavimab might be able to help treat Omicron in kidney transplant recipients who did not respond well to vaccination [23]. In a French study evaluating COVID-19 morbidity after the administration of tixagevimab–cilgavimab in 333 kidney transplant recipients, there was a significantly reduced risk of symptomatic COVID-19 and fewer COVID-19-related hospitalizations (including intensive care unit) compared to patients who did not receive this preventative treatment [27]. In a study of 67 immunocompromised individuals to test the real-world efficacy and safety of targeted COVID-19 treatment, SARS-CoV-2 infection was linked to no fatalities and a tolerable safety profile, despite inadequate post-vaccination immune responses [28].

The answer to the question of whether an Evusheld dosage of 300 mg is sufficient to prevent Omicron infection has not yet been determined. Benotmane I and colleagues [29] studied the ability of Evusheld to neutralize Omicron in a group of kidney transplant recipients who received the medication to prevent them from contracting the Omicron BA [29]. Serum samples were taken from 63 adult kidney transplant recipients who had received prophylactic injections of Evusheld (150 mg of tixagevimab and 150 mg of cilgavimab). According to the findings, the effects of Omicron were successfully inhibited 29 days after the injection in fewer than 10% of patients who were treated with Evusheld. According to these findings, a dose of 300 mg of Evusheld is probably insufficient to provide the desired level of neutralizing activity when administered in vivo.

In the current study, three of the six patients in the non-tixagevimab–cilgavimab group with a confirmed COVID-19 infection required ICU care, while the remaining three patients died from COVID-19 infection. In general, tixagevimab–cilgavimab reduces the risk of SARS-CoV-2 infection and COVID-19-related hospitalization in immunocompromised patients, with hazard ratios of 0.75 and 0.41, respectively [30]. Preliminary results from the TACKLE study indicated that the tixagevimab–cilgavimab group had a 51% lower risk of serious disease or death compared to the placebo group [31]. Additionally, tixagevimab–cilgavimab worked well to keep rheumatology patients taking rituximab from contracting a symptomatic SARS-CoV-2 infection [32]. In contrast, Stuver et al. found no indication of a neutralizing effect in hematologic patients who were administered tixagevimab–cilgavimab [33].

Despite the short duration of follow-up and the small number of patients, this study included a special subset of immunocompromised patients, namely HD patients. In the intervention group, we included patients with an anticipated increased likelihood of COVID-19 morbidity and lower vaccination efficacy. This study validated the short-term safety and humoral and clinical efficacy of tixagevimab–cilgavimab in this patient cohort, underlining the potential usefulness of this medicine in this group of patients. However, additional trials with a greater number of HD patients and longer follow-ups are necessary to corroborate these findings.

## 5. Conclusions

This study demonstrated the short-term safety and efficacy of tixagevimab–cilgavimab for COVID-19 preexposure prophylaxis in HD patients who should benefit from a global preventive strategy. The results of this trial support the use of a single dose of tixagevimab–cilgavimab for the prevention of symptomatic and severe COVID-19. These findings require more studies with more HD patients and longer follow-up periods.

## Figures and Tables

**Table 1:** Sociodemographic data and clinical characteristics for 73 HD patients included in the study.

Variable Mean ± SD, *n* (%), Median (Q1–Q3)	Received Evusheld (*n* = 43)	Did Not Receive Evusheld (*n* = 30)	*p*
Demographic Data			
Gender: Male Female	25 (58.1) 18 (41.9)	21 (70) 9 (30)	0.541
Age (years):	51.7 ± 15.32	35.2 ± 8.43	0.002 *
Smoking habit: Nonsmoker Smoker Ex smoker	34 (79) 3 (7) 6 (14)	27 (90) 3 (30) 0	0.388
Anthropometric measures:			
Weight (kg)	81.02 ± 21.96	75.3 ± 19.77	0.459
Height (m)	1.66 ± 0.24	1.56 ± 0.34	0.392
BMI (kg/m^2^)	27.8 (24.2–32.3)	26.3 (22.4–31.4)	0.522
Clinical characteristics of hemodialysis			
Duration of hemodialysis (years)	4 (2–7)	1.5 (1–2.625)	0.006
Therapeutic data			
Erythropoietin	26 (60.4)	24 (80)	0.077
Iron supplementation	25 (58.1)	21 (70)	0.302
Calcium supplementation	41 (95.3)	30 (100)	0.509
Alpha Calcidol	36 (83.7)	27 (90)	0.51
Calcimimetics	15 (34.9)	6 (20)	0.167
Antihypertensive drugs	14 (32.6)	9 (30)	0.817
Antidiabetic drugs	9 (20.9)	3 (10)	0.337
Immunosuppressive drugs	1 (2.3)	0	NA
Associated comorbidities			
Diabetes	9 (20.9)	3 (10)	0.664
Hypertension	23 (53.5)	21 (70)	0.488
Heart disease	7 (16.3)	0	NA
Liver disease	1 (2.3)	0	NA
Chronic respiratory disease	1 (2.3)	0	NA
Autoimmune disease	1 (2.3)	3 (10)	0.351

* *p* < 0.05. NA: not applicable.

**Table 2:** Previous COVID-19 infection and vaccination status.

Variable	Received Evusheld (*n* = 43)	Did not Receive Evusheld (*n* = 30)	*p*
Prior COVID-19 infection	13 (30.2)	6 (20)	0.706
COVID-19 vaccination	43 (100)	30 (100)	1
Type of COVID-19 vaccine: Sinopharm Sinovac Oxford AstraZeneca	6 (14) 33 (76.7) 4 (9.3)	6 (20) 24 (80) 0	0.566
Interval between COVID-19 vaccination and Evusheld (days)	170.8 ± 44.44	-	NA

NA: not applicable.

**Table 3:** Neutralization antibody titer scores over time in those with and without Evusheld use.

Evusheld	Timing					
	Baseline		After One Month		After Six Months	
	Median	Q1–Q3	Median	Q1–Q3	Median	Q1–Q3
Received	2	2–3	3	3–3	3	3–3
Not received	3	1–3	2	1–2	1.5	1–2

Notes: Q1 = 25th percentile. Q3 = 75th percentile. NAB in scored 1, 2, or 3 if it is <200, 200–800, or >800 AU/mL, respectively.

**Table 4:** COVID-19 infection after study enrollment.

Variable	Received Evusheld (*n* = 43)	Did Not Receive Evusheld (*n* = 30)	*p*
Total COVID infections (asymptomatic and symptomatic)	10 (23.3)	6 (20)	0.59
1. Asymptomatic SARS-CoV-2 infection	4 (9.3)	0	0.42
2. Symptomatic COVID-19 disease	6 (14)	6 (20)	0.63
3. COVID-19 related hospitalization	6 (14)	6 (20)	0.63
4. ICU admission for COVID-19	0	6 (20)	0.033 *
5. COVID-19 related death	0	3 (10)	0.036 *

* *p* < 0.05.

**Table 5:** Adverse effects of Evusheld in the study’s HD patients who received Evusheld.

Adverse Effects	Received Evusheld (*n* = 43) *n* (%)
Local pain and tenderness	2 (4.7)
Fever	1 (2.3)
Fatigue	1 (2.3)
Drowsiness	1 (2.3)
Fatigue, drowsiness, and dizziness	2 (4.7)

## Data Availability

The datasets generated during and/or analyzed during the current study are available from the corresponding author on reasonable request.

## Abbreviations

CKD	chronic kidney disease
COVID-19	coronavirus disease 2019
ESRD	end-stage renal disease
HD	hemodialysis
ICU	intensive care unit
PROVENT	Preexposure Prophylaxis of COVID-19 in Adults
SARS-CoV-2	severe acute respiratory syndrome coronavirus 2
WHO	World Health Organization
